# Healing by Laying on of Hands

**Published:** 1887-08

**Authors:** 


### HEALING BY LAYING ON OF HANDS.

It is against "the law" in New Hampshire, New York and various other sovereign States, to heal the sick by "the laying on of hands," as Jesus is reported to have done in the olden time. And the law was enacted by professed *Christians!* If this thing goes much further in this country in the interest of medico-creedism, honest people will be ashamed to be called Christians. It is a truly ludicrous condition of things, when we have on the one hand the Bible teaching us of healing in the manner specified above, that those who tenaciously adhere to the words of Scripture should so recklessly rebel against it. Certain of these very bigots, however, are of late adopting this simple method of cure, calling it "Christian Science!" when, the fact is, if they accomplish anything, it is through mediumistic power which they possess, mayhap unconsciously.

Then, again, there is another class who object to our healing mediums practicing, namely, the medical faculty, whose craft is in danger in consequence; and the latter are endeavoring to have laws passed in the different States, providing, under severe penalties, that none but "diploma" doctors shall practice. In twenty-nine States, we are ashamed to be obliged to say, they have succeeded. In others their endeavors have signally failed.

Even some among these selfsame doctors, who so persistently, as a body, attempt to obtain such a law, have individually, in many cases when they could not diagnose the disease of a patient, resorted to our healing and clairvoyant mediums without wishing it known that they were "physicians of regular standing." Then, after curing their patients, they have received the credit due alone to the medium. We know of many just such cases.

Another thing the general public are not fully cognizant of, but which they should be, is the fact that in this State alone there are many healing mediums, male and female, who have practiced for years with great success. We will cite one case, in this city, of a medium whom we have known for thirty years and over, Mrs. B. K. Little, controlled by Spirit John Dix Fisher, who was, before passing to the higher life, well known in Boston as one of the most competent medical advisers of his day. He was chiefly instrumental in founding the institution for the blind at South Boston. This spirit doctor also controlled, at different times, for quite a long period, Mrs. J. H. Conant, who was connected with this paper for over nineteen years, and we were cognizant of many wonderful cures in that time made by him through her agency.

To illustrate the power of the spirit over matter, we will cite merely one instance in the career of Mrs. Little, the truthfulness of which can be attested by her husband and others. Owing to her too frequent sittings she became herself an invalid, from the almost constant taking on of the unhealthy aura of her patients, until finally she was stricken with paralysis. The case was so bad that her life was despaired of, as she was obliged to lie in bed without the power of using her limbs. But Dr. Fisher assured the husband that he could cure her ; and he did. He would entrance her daily, take her bodily from the bed, walk her across the room for some time, and then replace her in the bed. When told repeatedly what had been done, she declared that it was an impossibility —that they could not make her believe she had been out of bed at all. But at length, after the Doctor had exercised her sufficiently, she began to have once more the use of her limbs, and ultimately recovered her health. She is now, or was when we last saw her, more robust than ever before. We make this statement without her knowledge or consent, therefore the reader will understand that it is not in any manner to be considered as an advertisement of the lady's healing powers by and through the instrumentality of our dearly loved friend, Spirit Dr. John Dix Fisher.

As practical evidence of what has been done for human good before the public eye by a magnetic healer, and as substantial reasons why other possessors of the same power should be left as free, legally, to operate with their beneficent gifts among the suffering as he was when on earth, we cite the following from the record of the life-work of Dr. J. R. Newton :

As long ago as 1858, Dr. Newton commenced practice in Cincinnati, O., as a public healer, and treated about one hundred a day with remarkable success. Be it said to his honor, he gave the credit to whom it belonged ; acknowledged the presence and recognized the invaluable assistance afforded him by his spirit guides and helpers. Here are a few of the thousands of cures performed by him in the early days of his practice :

Miss Catharine Johnson, Sixth street, blind for fifteen years, restored in fifteen minutes to read and work as well as when a child.

H. Oldham, Camden, Ohio., restored from paralysis and rheumatism in half an hour, to walk without limping.

Daniel Rice, near Kokomo, Ind., hip disease, confined to bed four months ; left his crutch and walked to hotel.

Maria Louisa Crane, Cincinnati, O., spine disease over two years, legs withered and drawn up ; five months previous to being cured could not be turned in bed, but lay in one position. Fully restored. It is not probable that a more wonderful cure has been for ages.

Miss Sarah Hinsey, Somerville, O., had not stepped on her foot for eight months ; with thirty minutes' operation left her crutches and walked to hotel.

Mrs. Bromwell, 293 George street, had lost all use of her limbs by spinal disease and weakness ; had not walked for eight months ; restored in twenty minutes, so as to walk about the house and out of doors.

George Bechtolds, Newport, Ky., daughter aged eight years, spine disease, had never walked ; with fifteen minutes' operating run about the room.

Miss Harriet Rall, daughter of Louis Rall, M. D., Cincinnati, could not speak plainly, and for five months had not spoken above the slightest whisper ; was perfectly restored, with clear pleasant voice as any one.

William Owen, corner Court and Western Row, inflammatory rheumatism. His entire flesh was so sore that he could not be touched without great pain. In fifteen minutes he arose from his bed cured, and walked nearly a mile.

Frances Harty, fourteen years old, Cincinnati, hip disease, walked on all fours if her crutch was taken from her ; cured in thirty minutes ; never used crutch afterward.

Jane Scott, Cincinnati, lame ankle, scarcely able to walk for twelve years ; made to walk without halt or limp in fifreen minutes.

Dr. Newton has passed to the higher life, but he is remembered in the grateful hearts of the many thousands he relieved of suffering and restored to health ; and that excellent book, "The Modern Bethesda," is a monument to his memory and his worth more enduring than marble. To that book we refer our readers as one highly instructive in the reliable information it gives regarding the natural gift of healing.

The experience of A. S. Hayward, magnetic healer of Boston, illustrates the treatment visited—where they have the power—by the opponents of improved remedial methods upon those who are in daily exercise of them for the benefit of humanity. This gentleman was prevented, in 1882, by the allopathic doctors of Saratoga Springs from giving magnetic treatment, under the alleged complaint of the people.

Mr. Hayward had visited this health resort annually for some fourteen years previously, and the citizens of the place, also sundry visitors, earnestly desired his treatment ; the allopathic doctors, however, wanted the practice for themselves, hence the attempted arrest of Dr. Hayward. The society which caused legal proceedings to be commenced, it is alleged, ceases to exist as a society, but the individual doctors still are watching their interests, and can enter a complaint, and cause trouble and expense to all clairvoyant and magnetic physicians, who may be in their county exercising their God-given healing gifts, at the request of the sick who desire and need their services.

Mr. Hayward has but recently been called upon to face the opposition of the Regulars in New Hampshire. According to the information furnished us, a party from Washington, D. C., in the employ of the United

States government, became much reduced and exhausted by too close application to his business, and was advised to seek restoration in the vitalizing air of the Granite State. The physician attending him there was puzzled to know what to do, and desired consultation with other physicians of his school; but the wife, as well as the sick man, wanted the magnetist in question, and sent to him to come at once to them in their distress. Mr. Hayward replied, saying that he would come to them as a friend, but not as a practitioner, as the laws of New Hampshire were such, as would impose fine and imprisonment upon him if he attempted to heal the sick in that State, but in deference to the earnest desire of these people, he complied, treating the patient successfully, and doing for him a work entirely outside of and beyond the range of the ancient methods of drug medication, upon which the State has seen fit to set its seal of approval. Here is a clear case where the relief of human suffering is regarded as a crime; and Mr. Hayward is rightly indignant that in the legitimate exercise of his calling, and at the earnest call of a stricken invalid, he was forced to assume the character of a law-breaker. Cases like this cannot fail to be provocative of thought in coming time, as to the gross injustice of all medical monopoly laws.

The present struggle over the "medical law" of Maine—which Gov. Bodwell vetoed, in which fearless action he was sustained by the Senate—is doing much good in educating the people up to a standpoint where they will discover, that nine-tenths of the causes operating to produce the passage of restrictive medical laws, have their spring in a determination on the part of the regular practitioners and their allies, to force the people "will ye nil ye," to employ them whether the patient has any confidence in their methods or not.—*Extracted from an article in Banner of Light, of July 9th.*

